# Effect of Specific Postural and Breathing Instructions on the Sagittal Alignment of the Spinopelvic Complex Before and After a Dedicated Muscle Strengthening Program: A Pilot Study in a Gymnast Population

**DOI:** 10.3390/jfmk11020171

**Published:** 2026-04-24

**Authors:** Camille Eyssartier, Pierre Billard, Patricia Thoreux, Christophe Sauret

**Affiliations:** 1 Arts et Métiers Institute of Technology, EPF Engineering School, Université Sorbonne Paris Nord, IBHGC–Institut de Biomécanique Humaine Georges Charpak, F-75013 Paris, France; 2 Fédération Française de Gymnastique, F-75010 Paris, France; 3 Grand Hôpital de l’Est Francilien, F-77100 Meaux, France; 4 Centre d’Investigations en Médecine du Sport, Hôpital Hôtel Dieu, Assistance Publique-Hôpitaux de Paris, F-75004 Paris, France; 5 Centre d’Etude et de Recherche sur l’Appareillage des Handicapés (CERAH), Institution Nationale des Invalides, F-75007 Paris, France

**Keywords:** spine, pelvis, hypopressive, gymnastics, low back pain

## Abstract

**Background**: Gymnasts are reported as a population at high risk of low back pain. The prevention and treatment of low back pain often rely on improving the effectiveness of deep stabilizer muscles through exercises that aim to reach spinal alignment and axial lengthening. However, the scientific evidence regarding the effect of the specific instructions used during exercises on the spinopelvic complex is still lacking. To address this gap of knowledge, the aim of this pilot study was to examine the effect of specific postural and breathing instructions (spine straightening, forced expiration and perineal contraction) on the sagittal alignment of the spine before and after fifteen weeks of a specific muscle strengthening program. **Methods**: Low-dose biplanar radiographic images were taken in neutral position and in the five specific postures before and after the program and associated skeletal 3D reconstructions were performed allowing calculation of sacral slope, pelvic tilt, lumbar lordosis and thoracic kyphosis. **Results**: Sixteen gymnasts completed the entire protocol and were included in the analysis. At the end of the program, most of the postures tested led to a decrease in sacral slope, an increase in pelvic tilt, a reduction in lumbar lordosis, and a decrease in thoracic kyphosis, but with varying efficiency; the condition combining spine straightening, perineal contraction and forced expiration appeared to be the most effective in influencing all parameters simultaneously. **Conclusions**: The results strongly encourage combining an instruction of spine straightening with instructions of both expiration and perineal contraction, which is information of interest for coaches, physiotherapists and medical professionals.

## 1. Introduction

Low back pain is highly prevalent in gymnasts [1,2], likely because of the high mechanical loads applied to the spine during training and competition [3,4,5]. To prevent and treat low back pain, hypopressive exercises are sometimes prescribed with the aim of improving reflexive activation of the deep trunk muscles without increasing intra-abdominal pressure. Although some studies have analyzed the effect of hypopressive exercises on lumbopelvic position, low back pain or deep trunk muscles activations [6,7,8], there is a lack of evidence regarding the effect of both individual and combined specific instructions (i.e., spine straightening, forced expiration, and perineal contraction) used during exercises on the actual sagittal alignment of the spinopelvic complex. Indeed, while the expected combined effect of the three instructions is to reach spinal alignment and axial lengthening of the spine, the real impact of each instruction or combination of instructions on the sagittal balance and the spinal axial length remains unknown. It is nevertheless important to understand the actual effect of these three combined instructions on posture, and also to observe how simplifications of the instructions affect these same postural elements. Indeed, simplifications could be either intentional on the part of the coach or the result of a misunderstanding or misapplication of the instructions by the athletes, and therefore the repercussions are currently unknown. As lengthening of the lumbar (resp. thoracic) spine is expected in relation to a reduction in lumbar lordosis (resp. thoracic kyphosis), measuring lumbar lordosis and thoracic kyphosis when executing these specific instructions would be very instructive. In addition, as pelvic retroversion (i.e., increase in pelvic tilt) enables transverse abdominal muscle activation [9,10] and contributes to a reduction in lumbar lordosis, the knowledge of the anatomical and positional parameters, in particular sacral slope and pelvic tilt, would be highly worthwhile for analysis.

These pelvic and spinal static parameters are difficult to measure accurately and only medical imaging techniques such as radiography can provide an effective response to this challenge. However, investigating multiple positions requires several images to be acquired, exposing the participants to potential high levels of irradiation, depending on the device used. Furthermore, certain imaging techniques require the person to be lying down, thus preventing the measurement of parameters of interest in a weight-bearing position. Fortunately, low-dose radiographic systems, such as the EOS system (EOS imaging, France), are nowadays available, enabling stereoradiographic images of the subject to be taken in frontal and lateral views simultaneously, with a low dose of irradiation and with the subject in a standing posture [11,12]. Additionally, these systems allow 3D reconstruction methods of the bones to be applied, especially for the pelvis and thoraco-lumbar spine [13] which enable calculation of spinopelvic parameters with great accuracy [14].

Thanks to such techniques, the aim of this study was to determine the effect of specific postural and breathing instructions (spine straightening, forced expiration and perineal contraction) on the sagittal alignment of the spinopelvic complex in a population of gymnasts, before and after fifteen weeks of a specific muscle strengthening program involving the postural and breathing instructions mentioned above. The potential effect of the strengthening program on low back pain prevention and/or treatment was outside the scope of the study. It was hypothesized that the sagittal alignment of the spine would change depending on the instructions and would evolve between before and after the training program.

## 2. Materials and Methods

### 2.1. Inclusion/Exclusion Criteria

The inclusion criteria were: being older than 12 years, competing gymnastics at national or international level, training more than fifteen hours per week, and having no pain or injury preventing gymnastics training for the next fifteen weeks. The presence or absence of low back pain was not a selection criterion for participation in the study. The exclusion criteria were: presenting an injury at the beginning of the study that would have prevented the participant from attending the whole training program, or receiving a contraindication to the program formulated by the medical doctor after both an interview and a medical examination.

### 2.2. Muscle Strengthening Program

Participants trained twice a week from thirty minutes to one hour for fifteen weeks and they were coached by a trainer from the French gymnastics federation specifically qualified for this program. The total duration of the program, the weekly number of sessions, and the session duration were chosen to both ensure a sufficient number of participants and to be integrated into the usual training of gymnasts, while being sustainable over time, so that most participants could complete the entire program, while still providing a reasonable opportunity to observe changes based on previous studies [15,16,17].

**Figure A1:**
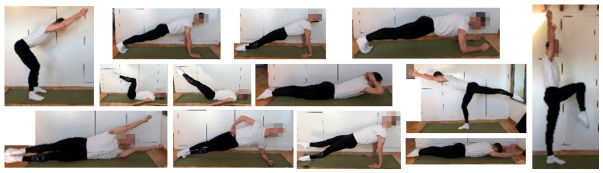
Photographs of the twelve exercises in which the three instructions (spine straightening, forced expiration, and perineal contraction) were implemented. During each session, between six and twelve exercises were performed. During the sessions, the exercises were repeated one to three times, each for durations ranging from 15 to 45 s.

The program itself consisted of twelve exercises (Figure A1, visible in Appendix A) combining hypopressive techniques with exercises such as the plank, the quadruped exercise, the superman exercise, etc. The specificity of the program consists—in order to ensure its hypopressive character—of using the following combined instructions across the twelve exercises performed: spine straightening, forced expiration and perineal contraction.

In order to minimize learning-related bias, all participants completed a familiarization session a few days before the initial assessment, during which the postures evaluated in the test were practiced, but without corrective feedback from the coach. More specifically, they were only told that spine straightening means becoming as tall as possible as if being pulled from the top of the head; perineal contraction is the action performed when holding back the urge to urinate; and forced expiration consists of blowing out all the air in the lungs, then holding the breath.

Only participants who attended at least 28 of the 30 training sessions proposed in the program were invited to undergo the final evaluation and were included in this paper.

### 2.3. Study Design and Data Collection

**Figure 1:**
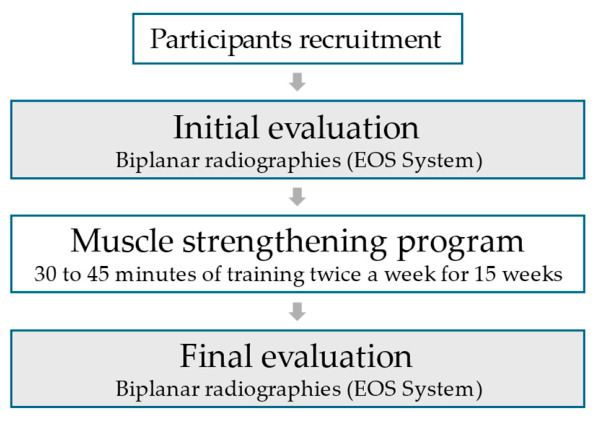
Flow chart of the study flow.

Two identical measurement sessions took place, one before the program (the initial evaluation) and one after (the final evaluation) (Figure 1). For each evaluation and each participant, a low-dose biplanar (face and lateral) radiograph (EOS system, EOS Imaging, Paris, France) in standardized neutral standing posture was first taken. Then, the participant was asked to execute the five following instructions while standing:

- Spine straightening (referred to as *straight*).
- Spine straightening while performing a forced expiration (referred to as *straight expi*).
- Spine straightening while performing a perineal contraction (referred to as *straight peri*).
- Forced expiration coupled with a perineal contraction (referred to as *peri expi*).
- Spine straightening while performing a forced expiration coupled with a perineal contraction (referred to as *straight peri expi*).

For each of these postures, a micro-dose biplanar EOS acquisition was taken. The rationale for using micro-dose acquisitions for these five postures instead of the low-dose acquisition was to limit the total radiation exposure. Indeed, low-dose acquisition was about 0.1–0.3 mSv [18] whereas a micro-dose acquisition was about 0.01–0.05 mSv [19,20], which would result for the full completion of the study in a total radiation dose lower than 1.1 mSv for each participant.

Each acquisition lasted about 20 s, and participants were encouraged verbally during this time so that they maintained the posture throughout. These specific postures were chosen in order to evaluate the effectiveness of the instructions and combinations of instructions in terms of the postural objectives previously stated: lengthening of the lumbar and thoracic spine in line with a decrease in lumbar lordosis and thoracic kyphosis and pelvic retroversion.

### 2.4. Data Processing

**Figure 2:**
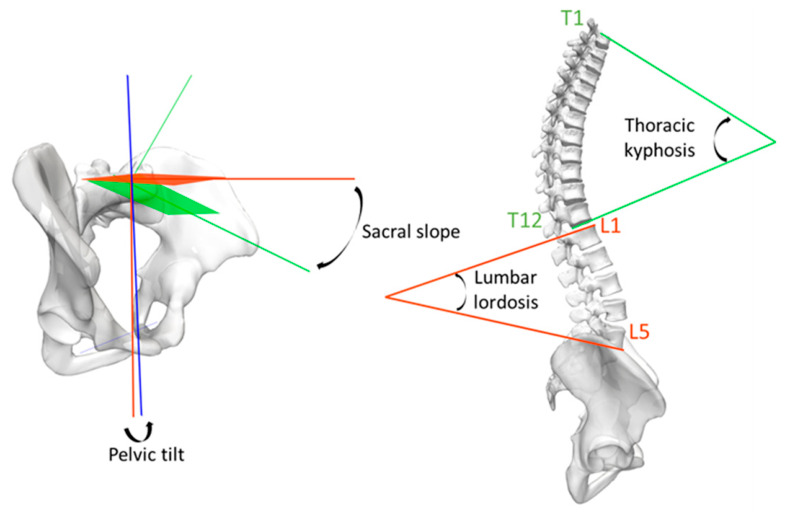
Illustration of the pelvic and spinal parameters considered in the analysis.

For each posture, 3D reconstructions of the pelvic bones and thoraco-lumbar vertebrae were performed [11,13] by operators certified for this process. The main pelvic (sacral slope, pelvic tilt) and spinal (lumbar lordosis, thoracic kyphosis) parameters characterizing the sagittal alignment of the spine were then extracted. For that purpose, a bone mesh was associated with a regionalization dataset for the bones of interest, which enabled the association of a 3D coordinate system to each bone and allowed the calculation of the parameters referenced in Table 1 and illustrated on Figure 2.

### 2.5. Statistical Analyses

The aims of the statistical analyses were:

(i) To compare postures with instructions with respect to the *neutral* posture during the initial and final evaluations;
(ii) To compare each posture during the initial evaluation to the same posture during the final evaluation;
(iii) To evaluate the effect of individual neutral curvature on changes during the different postures with instructions, for both initial and final evaluations.

For the comparison between postures at initial and final evaluations, statistical analyses were conducted using linear mixed models (LMM, type III, Sattherthwaite test method) to account for repeated and intra-subject variability. The postures were set as fixed effects variables and the participants as random effects grouping factors. Pre-specified contrasts were implemented to only compare the outcomes for each posture with instructions (i.e., straight, straight expi, straight peri, peri expi, and straight peri expi postures) against the reference condition (i.e., neutral posture) during the same evaluation (i.e., initial or final). *p*-values were adjusted using the Holm correction and the significance level was set at *p* ≤ 0.05. Regarding the effect of time (i.e., initial vs final evaluations), the same models were also applied, with posture and time included as fixed effects. Pre-specified contrasts were implemented to compare each posture at the initial evaluation with the corresponding posture at the final evaluation.

Finally, as the reduction in lumbar and thoracic spine curvature may be dependent on natural curvature in the neutral posture, Pearson’s r correlation coefficients were calculated for each parameter, comparing the value of these parameters in the neutral posture with the changes observed when different instructions were given. The results were interpreted as follows: *r* < 0.40 indicates a weak correlation, 0.40 ≤ *r* < 0.60 indicates a moderate correlation, 0.60 ≤ *r* < 0.80 indicates a strong correlation and *r* ≥ 0.80 indicates a very strong correlation. *p*-values were also calculated and the significance level was set at *p* ≤ 0.05.

All statistical analyses were performed using JASP version 0.19.3.0.3.

## 3. Results

### 3.1. Participants

Twenty-two gymnasts were included in the initial evaluation. They were involved in rhythmic gymnastics (10 participants), women’s artistic gymnastics (four participants) and men’s artistic gymnastic (eight participants). During the 15-week period, one men’s artistic gymnast sustained an injury during gymnastics practice affecting the full completion of the program and five rhythmic gymnasts were unable to complete the program due to a change in coaching staff and a shift in training priorities.

Hence, 16 gymnasts were finally included in the data analysis (five rhythmic gymnasts, four women’s artistic gymnasts and seven men’s artistic gymnasts). They were of 15 ± 2 years old on average (range: 12 to 21 years old); their masses were 53 ± 10 kg (range: 38 kg to 80 kg) and their heights were 1.55 ± 0.09 m (range: 1.40 to 1.73 m), resulting in BMIs of 22 ± 2 kg/m^2^ (range: 18 kg/m^2^ to 27 kg/m^2^).

### 3.2. Comparison of Instructions Effects

**Figure A2:**
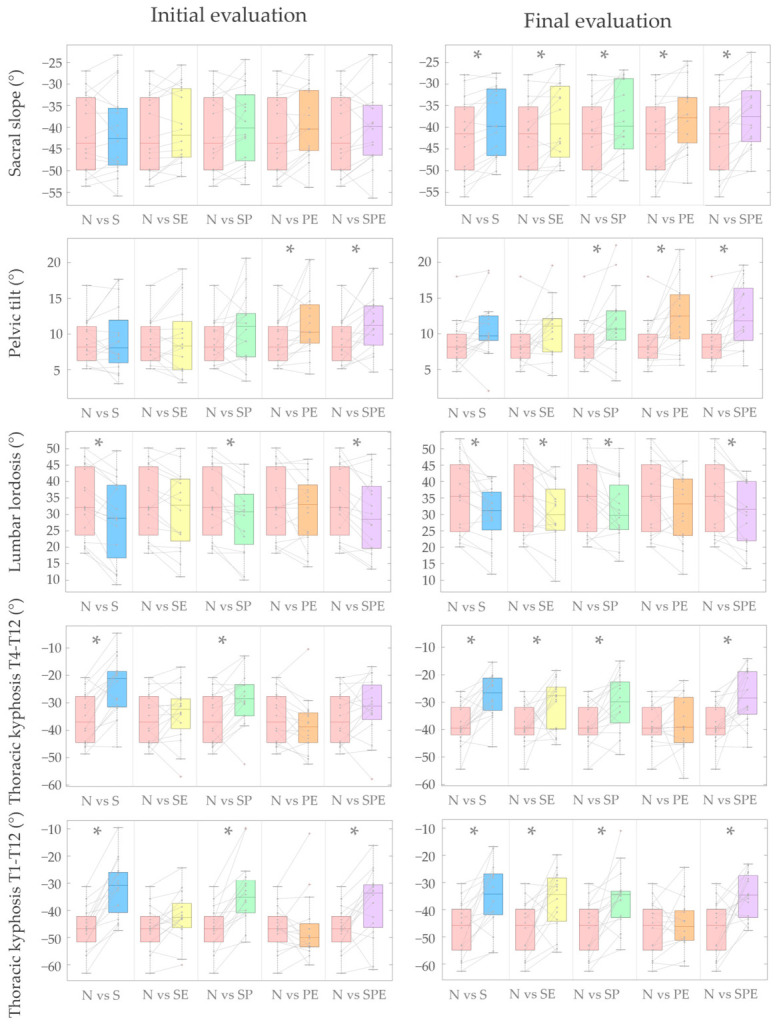
Whisker boxplots of the spinopelvic parameters for the different postures analyzed (*straight* (S), in blue; *straight expi* (SE), in yellow; *straight peri* (SP), in green; *peri expi* (PE), in orange; and *straight peri expi* (SPE), in purple) with respect to the *neutral* posture (N, in red) at initial (**left**) and final (**right**) evaluations. Regarding Whisker boxplots, the bottom and top of the boxes represent the first and third quartiles, respectively, and the horizontal line indicates the median. The ends of the whiskers show the maximum and minimum values. Red crosses indicate outliers. The grey circles show individual values, and the line connecting grey circles represents the individual changes between the postures compared. The grey stars (*) at the top indicate significant differences (*p* < 0.05) detected in specific contrast analyses.

Table 2 presents the mean values for the six specific postures studied (*neutral*, *straight*, *straight expi*, *straight peri*, *peri expi* and *straight peri expi*) and the *p*-values of the global ANOVA across all postures at both initial and final evaluations. Table 3 summarizes the average intra-individual variations between postures with instructions (*straight*, *straight expi*, *straight peri*, *peri expi* and *straight peri expi*) relative to the *neutral* posture and the *p*-value of the pre-specified contrasts analyzed. Figure A2, visible in Appendix B, illustrates the detailed individual results between the different postures with instructions relative to the *neutral* posture. Details of the LMM results are provided in the Supplementary Materials.

#### 3.2.1. Initial Evaluation

ANOVA performed on the LMM revealed a significant effect of posture on pelvic tilt, lumbar lordosis and both T4–T12 and T1–T12 thoracic kyphosis; but not on sacral slope (Table 2). Regarding pelvic tilt, pre-specified contrast analysis (Table 3) shows a significant increase only in the *peri expi* and *straight peri expi* postures (by approximately 2.5° on average). Lumbar lordosis was significantly reduced in the *straight*, *straight peri* and *straight peri expi* postures (by about 4.0 to 5.0° on average) but not in the *straight expi* and *peri expi* postures. Regarding thoracic kyphosis, the *straight* and *straight peri* postures resulted in a significant reduction in both T4–T12 and T1–T12 kyphosis, while the *straight peri expi* posture led to a reduction only in the T1–T12 kyphosis. The average intra-individual variations in thoracic kyphosis were the highest in the *straight* posture and decreased as additional instructions were introduced (i.e., *straight peri* then *straight peri expi*). Finally, none of the postures with instructions resulted in expected and significant changes for all the parameters studied.

#### 3.2.2. Final Evaluation

At the end of the program, ANOVA performed on the LMM results showed a significant effect of posture on all the parameters studied (Table 2). In contrast to the initial evaluation (Table 3), sacral slope was significantly increased in all postures with instructions compared to the *neutral* posture (ranging from 3.5° to 5.0° on average, depending on the posture); the *straight* posture showed the smallest increase, while the *straight peri expi* posture showed the greatest. Pelvic tilt was also increased in all postures with instructions compared with the *neutral* posture; however, this increase reached statistical significance only in postures involving perineal contraction (i.e., *straight peri*, *peri expi* and *straight peri expi* postures). For lumbar lordosis, all postures resulted in a reduction in lumbar curvature, which was significant for all postures except *peri expi*. Thoracic kyphosis (both T4–T12 and T1–T12) was also significantly reduced in all postures except *peri expi*. Finally, only *straight peri* and *straight peri expi* postures produced the expected significant changes across all parameters studied.

#### 3.2.3. Before vs. After Program Evaluations

Between the initial and final evaluations, two-way repeated measures ANOVA performed on the LMM results revealed a significant effect of time on sacral slope (*p* = 0.004) and pelvic tilt (*p* = 0.013) and pre-specified contrasts analysis only revealed a significant effect of time on sacral slope (*p* = 0.024).

### 3.3. Impact of Natural Curvature in Neutral Posture

Regarding the impact of neutral curvatures on their variation across the different postures studied, the correlation analysis showed at most moderate influences during the initial evaluation, which were disparate both in terms of postures and parameters (Table A1, visible in Appendix B). The highest correlation was obtained for pelvic tilt during the *straight expi* posture (r = 0.45), followed by sacral slope and T4–T12 thoracic kyphosis in the *peri expi* and *straight peri* postures, respectively (r = 0.41 and 0.40, respectively).

During the final evaluation, correlations were more pronounced and consistent, both in terms of postures and parameters. Indeed, moderate to strong correlations were found for sacral slope, lumbar lordosis and thoracic kyphosis (T1–T12 and T4–T12) (Table A1). More specifically, correlations for lumbar lordosis were found to range from 0.44 to 0.60 for four of the five postures studied: a strong correlation (r = 0.60) was found during the *straight* posture, and moderate correlations (0.44 ≤ *r* ≤ 0.56) were found for the *straight expi*, *straight peri* and *straight peri expi* postures. Regarding T4–12 thoracic kyphosis, a strong correlation was found during the *straight expi* posture (r = 0.68) and moderate correlations (0.44 ≤ *r* ≤ 0.56) were found for the *straight* and *straight peri expi* postures. For T1–T12 thoracic kyphosis, a strong correlation was found for the *straight peri expi* posture (0.77) and moderate correlations (0.52 ≤ *r* ≤ 0.54) were found for the *straight expi*, *straight peri* and *peri expi* postures. Finally, sacral slope was moderately correlated (r = 0.40 and 0.45) for the *straight* and *peri expi* postures.

All the correlation results are presented in Table A1, which is visible in the Appendix B section.

## 4. Discussion

### 4.1. Expected Effects

The objective of the present study was to determine the effect of specific postural and breathing instructions on the sagittal alignment of the spinopelvic complex before and after fifteen weeks of training of a specific muscle strengthening program. These instructions were designed to enable a decrease in lumbar lordosis and thoracic kyphosis, accompanied with a pelvic retroversion (resulting in a decrease in sacral slope and an increase in pelvic tilt). To meet this objective, the spinopelvic parameters (sacral slope, pelvic tilt, lumbar lordosis and thoracic kyphosis) of 16 gymnasts were determined in the neutral position and in the five specific postures of interest, based on EOS low-dose and micro-dose biplanar radiographs.

In theory, the expected effects of the different instructions would be:

- *Straight*: A decrease in both lumbar lordosis and thoracic kyphosis;
- *Straight expi*: A decrease in both lumbar lordosis and thoracic kyphosis, with a strengthening (compare to *straight* posture) of the reduction in lumbar lordosis induced by the diaphragm lift;
- *Straight peri*: A decrease in both lumbar lordosis and thoracic kyphosis, with a strengthening of the reduction in lumbar lordosis allowed by an increase in pelvic tilt accompanied by a decrease in sacral slope;
- *Peri expi*: A decrease in lumbar lordosis associated with an increase in pelvic tilt and a decrease in sacral slope;
- *Straight peri expi:* This was expected to provide the maximum reduction in both lumbar lordosis and thoracic kyphosis, combining the benefits of an increase in pelvic tilt (and a decrease in sacral slope) and diaphragmatic lift.

### 4.2. Initial Evaluation

With regard to the expected effects, prior to the program, the *straight* posture effectively reduced both lumbar and thoracic curvatures, without inducing any changes in pelvic parameters. The addition of a perineal contraction, (i.e., the *straight peri* posture), also led to expected reductions in both lumbar lordosis and thoracic kyphosis, and these changes were expectedly accompanied by an increase in pelvic tilt and a decrease in sacral slope. However, the modifications in pelvic tilt and sacral slope remained limited (approximately 2.0°), and the effects on lumbar and thoracic curvatures appeared slightly attenuated, whereas a strengthening of these effects has been anticipated. The addition of a third instruction (i.e., the *straight peri expi* posture) led to similar observations, with a further attenuation of the postural effects. At this stage of the program, combining multiple instructions appeared to reduce exercise performance. Moreover, the *straight expi* and *peri expi* postures failed to produce the expected effects.

From a practical perspective, the results of this pilot study suggest that the instructions should be introduced progressively throughout the first training sessions: first spine straightening, followed by the addition of a perineal contraction, and finally the inclusion of forced expiration. However, it appears inadvisable to introduce forced expiration in isolation, either combined solely with spine straightening or without spine straightening (i.e., *straight expi* and *peri expi* postures). Furthermore, individual results indicate that some gymnasts exhibited an increase in lumbar lordosis during the *straight* posture, contrary to the expected reduction. Therefore, it may be advisable to introduce a second instruction related to pelvic positioning from the outset, as already suggested in [21]. At this stage of the program, an instruction focusing on pelvic tilt may be more easily understood and effectively implemented than one based on perineal contraction.

### 4.3. Final Evaluation

At the end of the program, the isolated instruction of spine straightening (i.e., the *straight* posture) continued to produce reductions in both lumbar and thoracic curvatures. However, the pelvic parameters were now modified simultaneously, although pelvic tilt did not reach statistical significance. The introduction of forced expiration alone yielded overall results similar to those observed in the straight posture, although the reduction in thoracic curvature was slightly attenuated. The addition of a perineal contraction to spine straightening, as well as the combination of all three instructions, resulted in significant changes across all parameters, as expected. This time, however, combining the three instructions produced greater changes than applying one or two instructions simultaneously, highlighting the relevance of this combination once it has been adequately mastered. Finally, the *peri expi* posture appears to be of interest for pelvic positioning, but showed minimal effects on spinal curvatures.

In line with these findings, at the end of the program, nearly all gymnasts succeeded in reducing sacral slope compared to the *neutral* posture, regardless of the posture performed. This suggests that even without an explicit instruction for perineal contraction, a synergistic activation pattern may have been induced by the other instructions. Interestingly, the reduction in sacral slope was not systematically associated with an increase in pelvic tilt. Indeed, the pelvic bones complex is often considered rigid, and the sacroilliac joint is assimilated to a welded joint. However, recent research has shown that, although limited compared with most of the other joints of the human body, the sacroilliac joint does allow some degree of movement [22]. Therefore, given that the instructions provided throughout the program and during the radiographic acquisitions were not to tilt the pelvis backwards but to contract the perineum, the change in the sacral slope may have been achieved in some subjects with limited impact on pelvic tilt.

### 4.4. Before vs. After Program Evaluations

Through this muscle strengthening program, we observed a significant modification of the sacral slope in the neutral position as well as across all instructed positions, whereas no significant changes were found in the other parameters (pelvic tilt, lumbar lordosis, and thoracic kyphosis). Although the literature has not yet succeeded in demonstrating an effect of exercise programs [16], including hypopressive exercises [6], on lumbar lordosis and pelvic tilt, previously reported effects of exercise interventions on thoracic kyphosis [16] were not observed in our study. This discrepancy may be explained by the already high level of muscular conditioning in gymnasts, in contrast to the populations included in the referenced systematic review [16], which are generally older and either untrained or detrained. Moreover, an effect on sacral slope, despite the absence of changes in pelvic tilt, has not been previously reported. This may be explained by the fact that few studies have used outcome measures requiring radiographic imaging, thereby limiting the assessment of such parameters.

### 4.5. Impact of Natural Curvature in Neutral Posture

Finally, since the aim of the instructions was expected to induce a reduction in spinal curvatures, it was reasonable to assume that participants exhibiting the greatest curvatures in the *neutral* posture would be able to display the largest changes in these parameters across the different instructions. Our results showed that correlations were limited during the initial evaluation but were strengthened for lumbar lordosis and thoracic kyphosis (although not consistently across all postures) during the final evaluation. This suggests that neutral natural curvatures influence the magnitude of the response to the instruction, but that these effects only emerge once the muscles responsible for reducing these curvatures have been strengthened through the program.

### 4.6. Limitations

Although considerable care was taken in conducting this study, it nonetheless has certain limitations.

First, a control group would have been methodologically desirable, but its inclusion was not ethically acceptable, as it would have required exposing participants to ionizing radiation without any potential benefit. Indeed, the cumulative radiation exposure per participant in this study was approximately 1.1 mSv over the entire protocol (i.e., twelve stereoradiographic images: two acquired using low-dose settings and ten using micro-dose modalities). Although this is substantially lower than that associated with conventional digital radiography (approximately 0.5–0.7 mSv per image [20,23]), low-dose computed tomography (approximately 1.7 mSv per scan) [23], or standard computed tomography (approximately 8 mSv per scan [24,24]), it remains considerably higher than DXA examinations (approximately 0.005 mSv per scan [25]), which, however, do not provide equivalent measurements. Therefore, this level of exposure cannot be considered negligible in the absence of a clear potential benefit. Hence, baseline measurements, i.e., the results of the initial evaluation, were therefore considered to represent participants’ capacities in the absence of the intervention. Of course, gymnasts follow a sport preparation aside to the program and it is understandable to consider that this preparation is an uncontrolled cofounding factor. Given the already high amount of training that gymnasts typically undergo and the relatively short duration of the program, this potential effect is assumed to be negligible. Also, some gymnasts are relatively young and the intervention may have occurred during a growth spurt which would have compromised the results of the study. Here again, given the short duration of the program and the fact that not all participants could be concerned by a growth spurt simultaneously due to age differences in the cohort, we consider this effect to be negligible at the cohort level. However, we cannot rule out the possibility that it may have influenced one or two individuals in particular.

Second, the duration of the training program (15 weeks) and the training frequency (two sessions per week) can be considered limited, and the study would likely have benefited from a higher training volume to reveal differences more clearly. However, the chosen design was intended to remain close to a program that could be realistically integrated into gymnasts’ existing schedule. Also, increasing the duration would have increased the risk of withdrawal from the study due to the occurrence of injuries caused by gymnastics, preventing the program from being carried out in a controlled manner. In this case, we could consider that we exposed the participants to unnecessary ionizing radiation. Fortunately, in the present study, we only had one withdrawal from the study.

Third, although the training program was designed to address low back pain, this study did not directly assess its impact on this condition. Our focus was on spinopelvic adaptations during postures specific to the training program. These changes are thought to result from specific core muscles contractions, but we cannot directly investigate the effect of the program on low back pain prevention and treatment efficacy. Future studies should therefore investigate the effect of this program on low back pain prevention and treatment efficiency, which could be achieved without radiographic analyses and would allow for larger sample sizes.

Fourth, the effects observed during the final evaluation may be more related to an improvement in motor control than to an actual increase in muscular strength, but this cannot be ascertained. Nevertheless, this does not diminish the relevance of the study or its findings. Over a longer period, however, it can be expected that the strength of the targeted muscles would increase.

Fifth, regarding the radiographic assessments, the postures were not randomized. It is possible that, during the initial evaluation, some instructions were performed more effectively in later postures than in earlier ones, although a preliminary session was organized to limit this effect. This may represent a limitation of the initial evaluation, though not of the final evaluation, where participants were not expected to progress across the six tested postures. Moreover, the consistency of the results before and after the program supports the notion that any potential bias baseline had only a minimal impact.

Finally, it would have been interesting to examine how these instructions translated into different exercises (plank, superman, etc.); however, these positions were not compatible with the stereoradiographic system and would have significantly increased the number of images—and consequently the radiation exposure—which would have been ethically questionable. Technological developments allowing accurate prediction of spinopelvic parameters from a machine learning approach and based on non-irradiating measurements (or only one biplanar radiograph for personalization) could allow for such an objective in the future.

## 5. Conclusions

This pilot study aimed to evaluate the effects of specific instructions that are characteristics of hypopressive technique—spine straightening, forced expiration, and perineal contraction—on spinopelvic parameters before and after a core strengthening program incorporating these instructions. Changes in spinopelvic parameters, particularly reductions in sacral slope, lumbar lordosis, and thoracic kyphosis, were used to assess the effectiveness of spinal, abdominal, and pelvic floor muscle contractions. The effects of these instructions and the program were investigated in a population of gymnasts. The approach, with measurements based on 3D skeletal reconstruction from EOS biplanar radiographs, is original and enables precise quantification of the effects of individual instructions and their combinations on spinopelvic alignment.

The results highlight that the instructions induced measurable spinopelvic adaptations and that such changes were reinforced after a 15-week training program integrated into a realistic training schedule. Moreover, posture comparisons suggest that exercises combining all three instructions simultaneously are more effective in producing the expected changes across all spinopelvic parameters. However, performing all three instructions together can be challenging at the outset. Nevertheless, beginning with the spine straightening instruction alone can induce a counterproductive effect on the lumbar lordosis in some individuals. Combining spine straightening and perineal contraction, or alternatively with a simple posterior pelvic tilt, could represent a more suitable starting point. At the end of the 15-week program, the gymnasts achieved an automatic and synergistic integration of the three instructions, as evidenced by consistent sacral slope changes across all tested postures even when perineal contraction was not explicitly requested.

Although the limited sample size, program duration and volume, and absence of a control group require cautious interpretation, the consistency of the findings supports the robustness of the observed adaptations. Future research should confirm these results in larger cohorts and, above all, investigate the clinical relevance of this program, particularly its role in the prevention and management of low back pain.

## Figures and Tables

**Table 1:** Definition of the pelvic and spinal parameters considered in the analysis.

Parameter	Description
Sacral slope (°)	Angle between the horizontal plane and the plane of the sacral endplate.
Pelvic tilt (°)	Angle between the vertical axis and the line joining the centre of the sacral endplate to the midpoint of the bilateral hip axis.
Lumbar lordosis L1–L5 (°)	Angle between the cranial endplate of the vertebra L1 and the caudal endplate of the vertebra L5.
Thoracic kyphosis T4–T12 (°)	Angle between the cranial endplate of the vertebra T4 and the caudal endplate of the vertebra T12.
Thoracic kyphosis T1–T12 (°)	Angle between the cranial endplate of the vertebra T1 and the caudal endplate of the vertebra T12.

**Table 2:** Average values of spinopelvic parameters recorded during the initial and final evaluations across different postures in the gymnastics group. Results are presented as mean ± standard deviation. *p* represents the adjusted *p*-values comparing postures at initial and final evaluations.

Parameter	Evaluation	Neutral	Straight	Straight Expi	Straight Peri	Peri Expi	Straight Peri Expi	*p*
Sacral slope (°)	Initial Final	−41.6 ± 9.0 −42.4 ± 8.8	−41.3 ± 9.7 −38.9 ± 8.1	−39.9 ± 8.7 −38.5 ± 8.9	−39.5 ± 8.9 −38.3 ± 8.7	−39.2 ± 8.4 −37.8 ± 7.9	−39.6 ± 9.1 −37.3 ± 8.3	0.141 <0.001
Pelvic tilt (°)	Initial Final	8.8 ± 3.2 8.8 ± 3.2	9.2 ± 4.7 10.6 ± 4.1	9.3 ± 4.9 10.6 ± 4.1	10.8 ± 4.7 11.6 ± 4.9	11.5 ± 4.6 12.5 ± 4.5	11.5 ± 4.1 12.6 ± 4.5	0.005 <0.001
Lumbar lordosis L1–L5 (°)	Initial Final	33.7 ± 10.7 35.2 ± 10.9	28.7 ± 12.7 30.0 ± 8.8	31.2 ± 11.9 30.2 ± 9.4	29.0 ± 10.7 31.4 ± 10.5	31.4 ± 10.5 32.2 ± 10.4	29.6 ± 11.3 30.5 ± 9.8	0.025 0.042
Thoracic kyphosis T4–T12 (°)	Initial Final	−35.4 ± 9.5 −38.2 ± 7.3	−24.1 ± 10.8 −27.6 ± 7.8	−33.8 ± 9.5 −30.4 ± 9.0	−28.7 ± 9.8 −30.3 ± 9.8	−38.4 ± 10.1 −37.3 ± 9.8	−32.0 ± 11.2 −27.9 ± 9.8	<0.001 <0.001
Thoracic kyphosis T1–T12 (°)	Initial Final	−36.7 ± 8.1 −37.2 ± 9.8	−21.9 ± 10.5 −25.2 ± 12.2	−32.5 ± 8.0 −26.9 ± 11.0	−23.7 ± 11.7 −25.9 ± 10.9	−36.7 ± 12.0 −35.5 ± 9.6	−27.7 ± 12.7 −25.0 ± 8.2	<0.001 <0.001

**Table 3:** Summary of posture-related effects relative to the *neutral* posture during initial and final evaluations. Results are presented as mean ± standard deviation. Bold and green font indicate significant differences in pre-specified contrasts analysis. *p*-value (adjusted with Holm correction) are indicated between parentheses. Acronyms: SS: sacral slope; PT: pelvic tilt; LL: lumbar lordosis L1–L5; TK4: thoracic kyphosis T4–T12; and TK1: thoracic kyphosis T1–T12. Note that the increase arrow (↑) (resp. decrease arrow (↓)) corresponds to moving away from (resp. closer to) 0°.

		Straight	Straight Expi	Straight Peri	Peri Expi	Straight Peri Expi
Initial	SS PT LL TK4 TK1	↓ 0.3° ± 4.4° (0.79) ↑ 0.4° ± 2.9° (1.00) **↓ 5.0° ± 6.6° (0.01)** **↓ 11.3° ± 7.7° (<0.01)** **↓ 14.9° ± 7.4° (<0.01)**	↓ 1.6° ± 3.2° (0.26) ↑ 0.4° ± 2.6° (1.00) ↓ 2.5° ± 5.1° (0.26) ↓ 1.6° ± 8.2° (0.45) ↓ 4.3° ± 7.1° (0.14)	↓ 2.0° ± 4.5° (0.24) ↑ 1.9° ± 4.6° (0.10) **↓ 4.7° ± 5.3° (0.02)** **↓ 6.7° ± 8.3° (0.01)** **↓ 13.5° ± 8.3° (<0.01)**	↓ 2.4° ± 5.4° (0.15) **↑ 2.6° ± 3.9° (0.02)** ↓ 2.3° ± 6.9° (0.26) ↑ 3.0° ± 8.1° (0.30) ↓ 0.1° ± 9.5° (0.98)	↓ 1.9° ± 4.9° (0.24) **↑ 2.6° ± 3.5° (0.02)** **↓ 4.1° ± 6.9° (0.04)** ↓ 3.5° ± 9.6° (0.29) **↓ 9.0° ± 10.4° (<0.01)**
Final	SS PT LL TK4 TK1	**↓ 3.5° ± 3.4° (<0.01)** ↑ 1.8° ± 2.9° (0.09) **↓ 5.2° ± 7.9° (0.02)** **↓ 10.6° ± 8.8° (<0.01)** **↓ 12.0° ± 11.4° (<0.01)**	**↓ 3.8° ± 3.4° (<0.01)** ↑ 1.4° ± 3.7° (0.09) **↓ 5.0° ± 7.2° (0.02)** **↓ 7.9° ± 12.3° (<0.01)** **↓ 10.3° ± 12.7° (<0.01)**	**↓ 4.1° ± 4.5° (<0.01)** **↑ 2.8° ± 3.9° (0.01)** **↓ 4.1° ± 8.2° (0.05)** **↓ 8.1° ± 9.0° (<0.01)** **↓ 11.3° ± 12.5° (<0.01)**	**↓ 4.6° ± 4.5° (<0.01)** **↑ 3.7° ± 4.2° (<0.01)** ↓ 3.0° ± 6.8° (0.10) ↓ 1.0° ± 9.3° (0.68) ↓ 1.7° ± 9.8° (0.49)	**↓ 5.1° ± 4.0° (<0.01)** **↑ 3.8° ± 3.7° (<0.01)** **↓ 4.8° ± 5.8° (0.03)** **↓ 10.3° ± 10.9° (<0.01)** **↓ 12.2° ± 12.8° (<0.01)**

## Data Availability

The raw data are not readily available for ethical reasons (participants did not give consent). However, the original contributions presented in the study are included in the article and its appendix materials. Further enquiries can be directed to the corresponding authors.

## Supplementary Materials

The following supporting information can be downloaded at: https://www.mdpi.com/article/10.3390/jfmk11020171/s1, Table S1: detailed results of the LMM analyses.

## Appendix B

**Table A1:** Results of Spearman R (*p*-value) between the parameters in the neutral posture (SS: sacral slope; PT: pelvic tilt; LL: lumbar lordosis L1–L5; TK4: thoracic kyphosis T4–T12; thoracic kyphosis T1–T12) and the changes in these parameters between postures with instructions with respect to the neutral posture.

Evaluation	Postures	Parameters				
		SS	PT	LL	TK4	TK1
Initial	Straight	−0.09 (0.75)	0.19 (0.47)	−0.02 (0.95)	−0.23 (0.39)	−0.08 (0.76)
	Straight expi	−0.27 (0.32)	0.45 (0.08)	<0.01 (0.99)	−0.31 (0.24)	−0.29 (0.28)
	Straight peri	−0.27 (0.32)	−0.31 (0.25)	−0.26 (0.34)	−0.40 (0.13)	0.03 (0.92)
	Peri expi	−0.41 (0.12)	−0.16 (0.55)	−0.36 (0.17)	−0.35 (0.19)	−0.07 (0.81)
	Straight peri expi	−0.25 (0.36)	−0.24 (0.37)	−0.24 (0.37)	−0.32 (0.23)	−0.06 (0.83)
Final	Straight	−0.40 (0.12)	−0.22 (0.40)	−0.60 (0.01)	−0.53 (0.33)	−0.34 (0.19)
	Straight expi	−0.24 (0.38)	−0.39 (0.13)	−0.52 (0.04)	−0.68 (<0.01)	−0.54 (0.03)
	Straight peri	−0.29 (0.27)	−0.06 (0.29)	−0.56 (0.03)	−0.27 (0.31)	−0.54 (0.03)
	Peri expi	−0.45 (0.08)	−0.28 (0.29)	−0.38 (0.15)	−0.32 (0.22)	−0.52 (0.04)
	Straight peri expi	−0.35 (0.19)	−0.17 (0.53)	−0.44 (0.09)	−0.48 (0.06)	−0.77 (<0.01)
